# Immune damage mechanisms of COVID-19 and novel strategies in prevention and control of epidemic

**DOI:** 10.3389/fimmu.2023.1130398

**Published:** 2023-03-07

**Authors:** Yuting Sun, Bin Luo, Yueping Liu, Yuzhang Wu, Yongwen Chen

**Affiliations:** ^1^ School of Medicine, Chongqing University, Chongqing, China; ^2^ Institute of Immunology, People’s Liberation Army, Third Military Medical University, Chongqing, China

**Keywords:** COVID-19, SARS-CoV-2, t cell exhaustion, inflammation, kidney, lymph organs, immune damage

## Abstract

Caused by severe acute respiratory syndrome coronavirus 2 (SARS-CoV-2), coronavirus disease 2019 (COVID-19) has diverse clinical manifestations, which is the main feature of the disease, and the fundamental reason is the different immune responses in different bodies among the population. The damage mechanisms of critical illness by SARS-CoV-2 and its variants, such as hyperinflammatory response, a double-edged function of type I interferon, and hyperactivation of the complement system, are the same as other critical illnesses. Targeting specific immune damage mechanisms of COVID-19, we scored the first to put forward that the responses of T cells induced by acute virus infection result in “acute T-cell exhaustion” in elderly patients, which is not only the peripheral exhaustion with quantity reduction and dysfunction of T cells but also the central exhaustion that central immune organs lost immune homeostasis over peripheral immune organs, whereas the increased thymic output could alleviate the severity and reduce the mortality of the disease with the help of medication. We discovered that immune responses raised by SARS-CoV-2 could also attack secondary lymphoid organs, such as the spleen, lymphoid nodes, and kidneys, in addition to the lung, which we generally recognize. Integrated with the knowledge of mechanisms of immune protection, we developed a coronavirus antigen diagnostic kit and therapeutic monoclonal antibody. In the future, we will further investigate the mechanisms of immune damage and protection raised by coronavirus infection to provide more scientific strategies for developing new vaccines and immunotherapies.

## 1 Introduction

Among the four genera in the family *Coronavirinae*, the subfamily of *Coronaviridae*, alpha, and beta coronavirus have been transmitted into human beings. In contrast, gamma and delta coronavirus are circulated among animals. The infection of alpha coronavirus leads to mild symptoms similar to a common cold. In contrast, beta coronavirus leads to severer conditions, including severe acute respiratory syndrome (SARS), Middle East respiratory syndrome (MERS), and corona virus disease 2019 (COVID-19). There are more than 600 million COVID-19 cases and 6 million deaths worldwide. The significant characteristics of the coronavirus are as follows: (1) a variety of receptors are needed for the entrance of the virus in different genera into a multitude of hosts, and (2) the infection of the virus results in diverse clinical symptoms, from asymptomatic, mild, severe, to even mortal (1, 2). One significant feature is that, in some cases, the infection of SARS-CoV-2 is self-limited. Still, in other cases, the virus could infect endothelial cells, hypodermal cells, and epithelial cells, causing the lesions of many tissues and organs. Its self-limited infection process and natural history reveal that COVID-19 is a typical immune-related disease that determines whether the immune response determines the viral clearance or the pathological damage. Therefore, immunological prevention and control are the most fundamental methods to combat the pandemic, whereas distinguishing the mechanisms of immune protection and damage after SARS-CoV-2 infection in the human body is the precondition of immuno-diagnosis, immuno-prevention, and immune therapy. Because SARS-CoV-2 is a sort of cytopathic virus, the infection itself causes primary injury, and the damaging immune responses lead to secondary injury. This article discusses the specific characteristics of the virus and its unique mechanisms of immune damage to provide scientific fundamentals for diagnosing, preventing, and treating COVID-19.

## 2 The antigen and variants of SARS-CoV-2

### 2.1 The antigen of SARS-CoV-2

SARS-CoV-2 is an enveloped single-stranded positive-sense RNA virus spherical in shape with a diameter of approximately 120 nm and has 9- to 12-nm-long characteristic spikes on the surface. On the basis of phylogeny and genomic structure taxonomy, SARS-CoV-2, along with earlier identified SARS-COV and MERS-COV, belongs to the coronavirus beta genus (3). The SARS-CoV-2 genome contains 14 open reading frames (ORFs), including one large ORF that encodes two large polyproteins (ORF1a and ORF1ab) and 13 small ORFs that encode viral structural proteins and other polypeptides, including S antigen, N antigen, M antigen, and E antigen.

S antigen, split into S1 and S2 by FURIN protease, is a transmembrane protein outside the virus. Virus utilizes transmembrane protease, serine 2 (TMPRSS2), as the primer of the S protein. The S1 subunit promotes the combination of viral particles and host cells by binding to the angiotensin-converting enzyme 2 (ACE2) receptor (4). N antigen locates mainly in the endoplasmic reticulum and Golgi apparatus, combines viral RNA, and participates in virus replication and virus–anti-host immune response. M antigen combines coronavirus RNA, determines virus RNA replication, and participates in virus assembly and budding. E antigen plays a vital role in regulating the maturation of viral particles (5).

In the human body, S antigen is characterized by activating follicular helper T (Tfh) cells, which mainly triggers humoral immune response. The M and N antigen primarily activates Th1 and Th17 cells, which mainly triggers cellular immune response. The immunological characteristics of E antigen are rarely investigated (6). The development of vaccines and therapeutic antibodies is mainly underpinned by the protective immune reaction triggered by protective antigens. The appropriate antigens (immunogens) selection is essential, but the protective immune response based on the epitope’s specificity is more crucial. Such epitope-specific immune responses have already been discovered in the infection cohorts among the population. The immunogen design hopes to produce better vaccines, therapeutic antibodies, or immune cells.

### 2.2 SARS-CoV-2 variants and the immune escape

Under the pressure of the host’s immune system, SARS-CoV-2 mutates constantly. According to the transmissibility, the virulence, and the possibility of the variants breaking the immune barriers, the World Health Organization (WHO) has classified it into “variants of concern” (VOC) and “variants of interest” (VOI). Up to now, WHO has already identified five VOC variants, namely, alpha variant (B.1.1.7), beta variant (B.1.351), gamma variant (P.1), delta variant (B.1.617.2), and omicron variant (B.1.1.529). Many variations in epitopes of antibodies and T cells are taking place in all these variants, which could, to different degrees, help to escape the immune protection of the host on the original virus strain and theoretically lead to a wider prevalence of the disease. SARS-CoV-2 variants and their mutation numbers are listed in **Table 1**. A phylogenetic tree showing 3,064 genomes sampled between December 2019 and January 2023 is available on Nextstrain website (https://nextstrain.org/ncov/gisaid/global/all-time), which can help us better understand changing patterns of different lineages.

**Table 1:** SARS-CoV-2 variants and their mutation numbers.

Variants	Mutation numbers
Alpha (B.1.1.7)	20
Beta (B.1.351)	17
Gamma (P.1)	22
Epsilon (B.1.429)	10
Lota (B.1.526.1)	17
Delta (B.1.617.2)	18
Omicron (B.1.1.529)	42
P.2	10
P.3	20
B.1.618	16
B.1.526. 2	18
B.1.617.1	15
B.1.617. 3	14
Total	239

In addition to D614G mutation, alpha variants S protein carries mutations in other eight points: HV69-70del, Y144del, N501Y, A570D, P681H, T716I, S982A, and D1118H (7). The S protein D614G mutation could open the RBD structure and enhance the FURIN protease’s ability to split the S protein, promoting the more efficient combination of S protein and ACE2 receptor (8). Notably, N501Y mutation also increases the combination of S protein and ACE2 receptor (9). Therefore, these mutations enhance the transmissibility, infection hospitalization rate, and mortality of alpha variants. beta variants mainly has 8 amino acid mutations, namely, L18F, D80A, D215G, LAL242-244del, K417N, E484K, N501Y, and A701V, among which N501Y, K417N, and E484K are located in the RBD of the virus (8). N501Y mutation enhances the transmissibility of beta variants, whereas E484K mutation enhances its immune escape (10). Gamma variants mainly have L18F, T20N, P26S, D138Y, R190S, K147T, E484K, N501Y, H655Y, T1027I, V1176F, and S681H mutations in S protein. Unlike alpha and beta variants, K417T and L18F mutations enable the gamma variants to escape antibody neutralization in the host (11, 12). Delta variants were first discovered in India. Virus S protein mainly has 10 amino acid sites of point mutations: T19R, G142D, 156del, 157del, R158G, L452R, T478K, P681R, D950N, and S417N. Among them, three mutations, namely, L452R, E484Q, and P681R, are located in RBD, significantly reducing or even eliminating the ability of variants S protein binding to neutralizing antibodies. Moreover, the P681R mutation increases the splitting of S1/S2 to enhance the virus infection to target cells (12). The newly discovered omicron variants have 32 amino acid sites of point mutation, and the three mutations, namely, T478K, E484A, and Q493R, are specific to omicron variants. N501Y and Q493R mutations increase the affinity of omicron variants combining to ACE2 receptor. Therefore, the spread of omicron variants is far quicker than any other strains (13).

### 2.3 Vaccine protection on SARS-CoV-2 variants

Up to now, the investment and the effectiveness of the development of SARS-CoV-2 vaccines exceed any other infectious diseases in the past. Many countries have pinned their hopes on using vaccines to end the pandemic. More than 200 candidate vaccines based on traditional and new vaccine platforms are being investigated. Eight kinds of vaccines, including inactivated, adenovirus vector, mRNA, and recombinant subunit, are widely used in many countries. However, constantly emerging variants pose challenges to its effectiveness.

To inquiry whether inactivated vaccines have immune protections on variants, a virus neutralization assay was carried out on the serum of the vaccinators of the BBIBP-CorV from China Biologic Beijing Institute of Biological Products, Sinopharm Group, and the Corona Vac from Sinovac, Beijing, Ltd. It was discovered that geometric mean titers of BBIBP-CorV serum on alpha variants were basically in line with that on the wild strains. Still, the neutralization effectiveness of these serums on beta variants was significantly reduced or even disappeared (14). Moreover, Ali et al. discovered that, compared with other variants, omicron variants could easier break the immune defense induced by BBIBP-CorV (15). The effectiveness of serums from the recipients of vaccine Corona-Vac is slightly lowered on alpha and beta variants but completely disappeared on gamma variants, indicating that gamma variants escape the immune protection by vaccine Corona-Vac (14). The type 5 human adenovirus vector vaccine Ad5-nCoV developed by Academy of Military Medical Sciences-CanSino Bio, monkey adenovirus vector vaccine AZD1222 developed by Oxford-AstraZeneca, and Russian human adenovirus vaccine Sputnik V are now widely used adenovirus vaccines in the globe. The research found that, except for slightly decreased neutralization effectiveness on beta variants, the Ad5-nCoV vaccine still maintained its ability on other variants, which suggests a good protection outcome (16). Studies on vaccine AZD1222 showed that the neutralization efficacy on alpha variants decreased to 70.4% (17). However, the research by Wall et al. revealed that AZD1222 still keeps its protection on other variant types, including delta variants (18). However, the study on vaccine Sputnik V showed that the neutralization efficacy of its antiserum produced by this vaccine on alpha and beta variants is somewhat inferior (19).

Pfizer and BioNTech developed the BNT162b2 vaccine, and Moderna developed the mRNA-1273 vaccine, which are the two mRNA vaccines urgently approved on the market. The research discovered that the neutralization efficacy of the BNT162b2 vaccine on alpha and gamma variants lowered three to six times compared with the wild strains, but it lowered more than 40 times for beta variants. Another study pointed out that BNT162b2 vaccinators could be re-infected with delta variants, which suggested that BNT162b2 could lose long-term protection on delta variants (20). Recently, an assessment of the mRNA-1273 vaccine discovered that three doses of vaccination could effectively prevent the infection of delta variants, but its effectiveness on Omicron is rather low (21). Similarly, on the serum of 12 cases of BNT162b2 vaccinators, the neutralization efficacy of its antibody on omicron has lowered about 40 times (22). As for the heterologous immunization plan, two doses of mRNA-1273 vaccination followed by BNT162b2 enhancer could improve the neutralization effect of the vaccinator’s serum on omicron variants (23). ZF2001 is the recombinant subunit vaccine developed by George F Gao academician team, Institute of Microbiology, Chinese Academy of Sciences, and Anhui Zhifei Longcom Biopharmaceutical Co., Ltd. The tandem repeat dimer of S protein RBD is regarded as the antigen, and aluminum hydroxide is added as the adjuvant. On the basis of the large-scale adult cohort, at least 6 months after the complete vaccination, ZF2001 was proven safe and could effectively prevent symptomatic or even severe COVID-19 (1). Recently, the researchers discovered that antibody titration significantly increased after the third homologous (inactivated vaccines) or heterologous vaccination, and antibody positive rate reached at least 75% (24). Therefore, promoting and popularizing vaccine booster injections, either homologous or heterologous, is an effective means to prevent SARS-CoV-2 transmission, especially for the omicron variant strain.

With four omicron subtype strains (BA. 5, BQ. 1, BF. 7, and XBB) circulating globally, the impact of these subtype strains on the protective effects of vaccines has been a hot topic. A recently published research reported that neutralization of BQ.1, BQ.1.1, XBB, and XBB.1 by sera from vaccinees and infected persons was markedly impaired, even including sera from individuals boosted with a WA1/BA.5 bivalent mRNA vaccine, indicating that BQ and XBB subvariants present serious threats to current COVID-19 vaccines, render inactive all authorized antibodies, and may have gained dominance in the population because of their advantage in evading antibodies (25, 26). However, a well-accepted finding is that vaccine efficacy is substantially greater against severe disease than against any infection (27). This effect could be because protection against severe disease is mediated not only by antibody responses, which might be relatively short lived for some vaccines, but also by memory responses and cell-mediated immunity, which are generally longer lived (28, 29).

### 2.4 The impact of SARS-CoV-2 variants on the antigen-detection diagnostic kit

Shortly after the outbreak of COVID-19, viral diagnostic measures were deployed, including viral nucleic acids, viral antigens, or serological tests. This served to supplement the common disease signs and symptoms of COVID-19 of cough, fever, and dyspnea because all are commonly seen during seasonal upper respiratory tract infections, highlighting the importance of precise diagnostic tests to affirm SARS-CoV-2 infection. However, with the ongoing occurrence of SARS-CoV-2 variants, concerns grew regarding the accuracy of antigen-detection diagnostic kits developed to detect the original virus strain. Research reported that relative to the original D614G variant, there was a 10-fold loss of detection for the delta and alpha variants in specific antigen-detecting rapid diagnostic tests, a reduction above the threshold required to isolate the viable virus (30). However, beta and omicron variants did not lose detection capacity. As the new VOC arises, successful contact tracing requires continuous monitoring of antigen-detecting rapid diagnostic tests.

## 3 Novel immune damage mechanisms of COVID-19

Immune damage mechanisms are essential guidelines for clinical treatment and immune prevention. The existing studies revealed that SARS-CoV-2 infection not only triggered the hyperinflammatory response, delayed secretion of type I interferon (IFN-I), and hyperactivation of the complement system common in other pathogen infections but also directly infected the host’s innate immune cells and secondary lymphoid organs, which, in turn, caused immune exhaustion. Among the aged and immunosuppressed populations, acute T-cell exhaustion resulted in uncontrolled infection, clinically manifested as critical illness or even death. The details are depicted as follows.

### 3.1 Innate immune system damage by SARS-CoV-2 infection

#### 3.1.1 Hyperinflammatory response

The innate immune system mainly consists of innate immune cells such as mononuclear macrophages and neutrophils and their secreted cytokines, which is one of the first defensive lines of the host against virus infection. Unlike the influenza A virus H3N2, human rhinovirus, and respiratory syncytial virus, which invades the respiratory system, SARS-CoV-2 could infect not only the human type I alveolar cells but also the innate immune cells such as mononuclear-macrophages, neutrophils, and dendritic cells. SARS-CoV-2 infection could give rise to the accumulation of many cytokines (IL-6, TNF-α, M-CSF, IL-1β, and IL-10) and chemokines (MCP-1, IL-8, and CXCL-10) in serum and bronchoalveolar lavage fluid. Although its intensity is lower than the “cytokine storm” of aseptic sepsis, these cytokines and chemokines could increase the capillary permeability, accumulate the alveolar fluid, and influence the ventilation function, resulting in capillary leakage syndrome and multiple organ dysfunctions in critical patients. The manifestations include increased capillary permeability, edema, and acute respiratory distress syndrome. Therefore, a hyperinflammatory response is regarded as the indicator of poor prognosis of COVID-19 disease progression (31). The early usage of tocilizumab to block IL-6/IL-6R pathway activity and cytokine storm has been applied in patients with severe COVID-19 and received positive outcome (32). It needs to be pointed out that cytokines are various in different patients and at different time points of the same patients, and precise blockade may receive a better consequence.

#### 3.1.2 Delayed secretion of type I interferon

IFN-I is mainly produced by innate immune cells. It suppresses virus replication and therefore plays a crucial role in anti-virus immunity. The research discovered that the SARS-CoV-2 virus protein could inhibit the expression of several key molecules that regulate the IFN-I gene (Ifn-I) transcription pathway. For example, SARS-CoV-2 M protein could block the activation of interferon-stimulated genes (ISGs) and then inhibit the transcription of the host’s *Ifn-I* gene (33). Virus NP protein could inhibit the key signal transducer molecules downstream of the retinoic acid–inducible gene-I pathway and antagonize the IFN-β production. Moreover, the overactivation of the IFN-I signal pathway also contributes to the delayed secretion of interferon in patients with severe COVID-19 (34). SARS-CoV-2 MicroRNA (miRNA) SCV-miR-ORF1ab-1-3p and SCV2-miR-ORF1ab-25p play a role in immune escape by targeting many genes in the IFN-I signal pathway (35). It should be pointed out that IFN-I plays a protective role in the early phase of the disease but plays a damage role in the late phase. Precise use of IFN-I would receive better treatment outcomes.

#### 3.1.3 Over-activation of the complement system

Complement constitutes an important part of the innate immune system. Its appropriate activation may lead to the phagocytosis and lysis of invaded pathogens. Still, the overactivation may intensify the inflammatory response, leading to the injury of lung and epithelial cells, the microangiopathy, and thrombogenesis, resulting in a multiorgan failure in patients (36). For example, complement C3a, C5a, and sC5b-9 are deposited in alveolar type II cells of patients with COVID-19. In addition, C5a-C5aR1 could activate neutrophils and mononuclear cells to secret inflammatory factors, which form the hyperinflammatory response, whereas anti-C5aR1 monoclonal antibodies could suppress acute lung injury in patients with severe COVID-19. Therefore, blocking C5a-C5aR1 is an effective new strategy in severe COVID-19 treatment (37).

#### 3.1.4 The hyperactivation of NLRP3 inflammasomes is associated with COVID-19 severity

The NLRP3 inflammasome is an essential component of innate immune systems that plays a crucial role in controlling virus infection. SARS-CoV-2 infection could trigger the activation of NLRP3 inflammasome to release IL-1β, IL-18, and gasdermin D and, consequently, damage to lung tissue in patients with COVID-19, suggesting the dysregulation of NLRP3 inflammasome might contribute to the severity of COVID-19. Sefik et al. reported that SARS-CoV-2 infected human lung-resident macrophages to activate NLRP3 inflammasomes, thereby contributing to the hyperinflammatory state of the lungs (38). Toldo et al. showed that the lung sections from patients with fatal COVID-19 who had died of cardiopulmonary arrest expressed a significantly high level of NLRP3 inflammasome molecules (39). Further studies demonstrated that SARS-COV-2 encoding ORF3a, ORF-3b, N, and E antigens, respectively, can activate the NLRP3 inflammasomes (40). Therefore, target NLRP3 inflammasomes implicate a promising therapeutics to deal with COVID-19.

### 3.2 Adaptive immune system damage by SARS-CoV-2 infection

#### 3.2.1 Lymphopenia

The protective effect of adaptive immunity on the body is accomplished mainly by T cells and neutralizing antibodies. T-cell immunity plays a crucial role. The virus S protein-specific CD3^+^/Granzyme B^+^/perforin^+^ cytotoxic T lymphocytes (CTL) could be detected 2 days before the symptom onset in patients with COVID-19. Moreover, among patients with mild/asymptomatic or convalescent COVID-19, CD45RA^+^/CCR7^−^ memory T cells could also be discovered, which could resist SARS-CoV-2 reinfection (41). In some patients with COVID-19, a high effective anti–SARS-CoV-2 A2/S_269-277_HLA-A*02:01 and NP_105-113_-B*07:02 epitope-specific CTL has been recently found, which could antagonize infection of many virus variants **(** 42, 43). However, in the early stage of the disease, the white blood cells in the peripheral blood of patients with COVID-19 may be commonly normal or decreased. The lymphopenia may develop in 50%~83% of severe patients who declined total counts of lymphocytes. Further studies revealed that inflammatory factors could directly induce T cells apoptosis or pyroptosis, also known as inflammatory cell death, especially for the high antiviral activity IFN-γ^+^/TNF-α^+^/IL-2^+^/granzyme B^+^/CD4^+^ T cells and memory CD3^+^/CD45RO^+^/CD4^+^ T cells in the body with their quantities severely reduced. Therefore, lymphopenia is the critical factor for poor prognosis in patients with severe COVID-19 (33, 44).

#### 3.2.3 Acute T-cell exhaustion

Apart from lymphopenia, part of the acquired immune system damage, patients with severe COVID-19 are also accompanied by acute function exhaustion of T cells. Inhibitory receptor molecules, such as PD-1, TIM-3, and LAG-3, are highly expressed in CD3^+^ T cells in peripheral blood mononuclear cells of patients with severe COVID-19 induced by acute SARS-CoV-2 infection (45, 46). However, the frequency of NKG2A^+^/PD-1^+^/CTLA-4^+^/TIGIT^+^ exhaustion CTL in dead patients or patients with severe COVID-19 is significantly higher than in moderate/mild patients, suggesting that it is associated with patients’ poor prognosis (47). Subsequent single-cell RNA sequencing (scRNA-seq) revealed that T cells in patients with COVID-19 have exhaustion characteristics, including the expression of tissue-resident and memory phenotype (ZNF683^+^ and ITGAE^+^); high expression of inhibitory molecules PD-1, TIM-3, HAVCR2, LAG3, and CTLA-4; high expression of proinflammatory factors CD70, COTL, and HMGB1; and stress-related molecules HSPD1, HSP90AA1, and BIRC5 (48). It is indicated that SARS-CoV-2 triggers immune escape by inducing acute T-cell exhaustion in patients with severe COVID-19.

**Figure 1:**
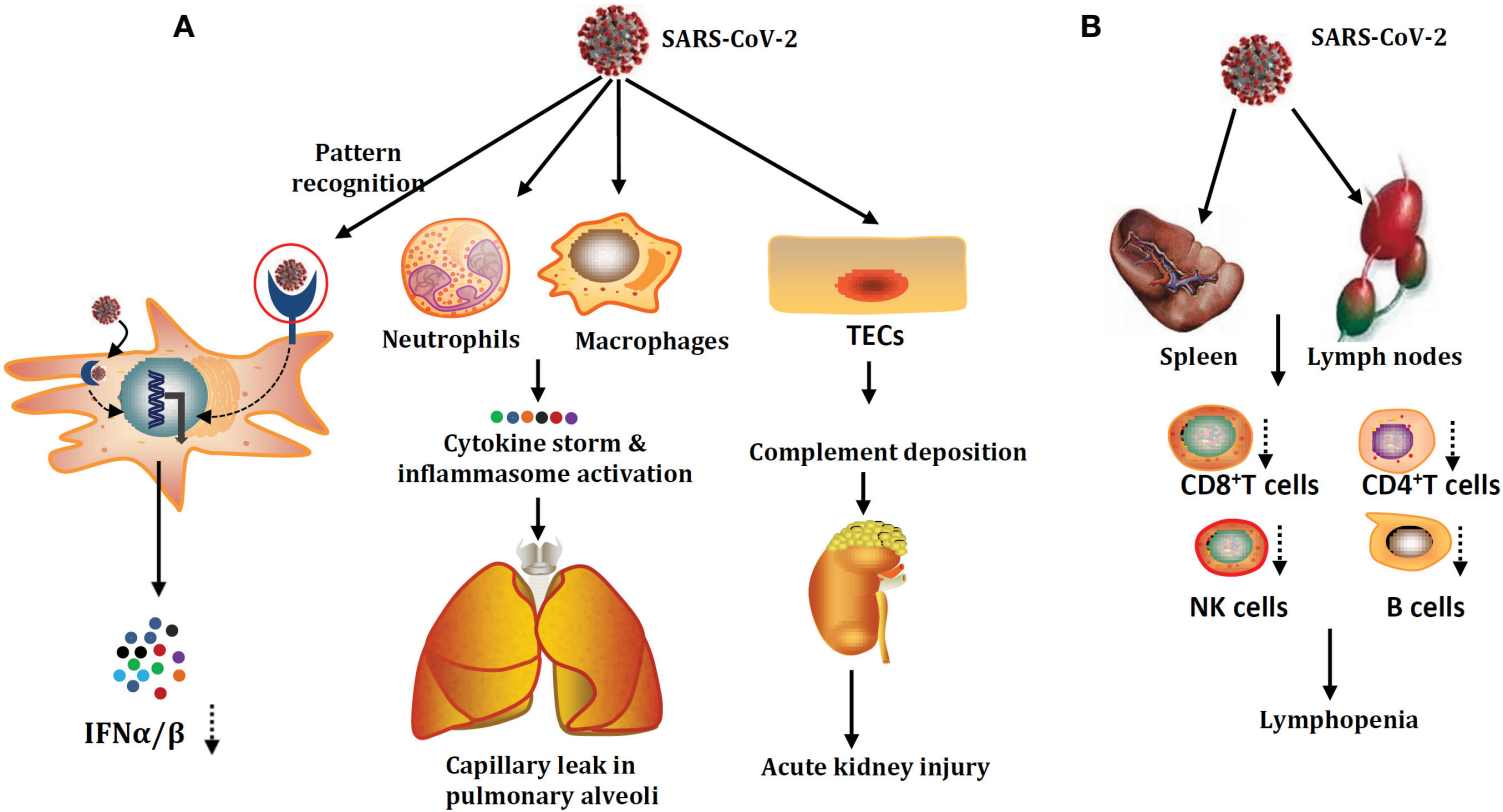
SARS-CoV-2 damage mechanisms on the innate immune system **(A)** and adaptive immune system **(B)**. SARS-CoV-2 could infect not only human type I alveolar cells but also the innate immune cells and tubular epithelial cells (TECs). SARS-CoV-2 infection could give rise to the accumulation of cytokine storms and hyperactivation of inflammasome. Apart from lymphopenia, the critical factor for poor prognosis in patients with severe COVID-19. Part of the acquired immune system damage, patients with severe COVID-19 are also accompanied by acute function exhaustion of T cells.

The damage to the innate immune system and acquired immune system by SARS-CoV-2 infection are summarized in **Figure 1**.

## 4 Novel immune damage mechanism of COVID-19 revealed by our team

Targeting human immunity, our team revealed the severe damage mechanisms of SARS-CoV-2 infection from the perspective of immune homeostasis to provide theoretical and technological support for preventing and controlling COVID-19.

### 4.1 The first in the world to discover that acute SARS-CoV-2 infection leads to “acute T-cell exhaustion” in severe patients

Lasker prize laureate Jacques Miller discovered 70 years ago that cytotoxic T cells are the main cells in the clearance of the virus. Two decades ago, Nobel Prize owner Rolf. M. Zinkernagel discovered that CTL is exhausted in chronic and persistent infections, resulting in the protracted course of the disease. Chronic exhaustion refers to the concept that, because of the long and persistent stimulus of the antigen, T cells gradually lose their ability to secrete IL-2, TNF-α, IFN-γ, and perforin; meanwhile, negative regulatory factors such as PD-1, TIM-3, CTLA-4, and TIGIT are highly expressed, which lead to exhaustion and anergy of CTL (49). Our team scored the first in the world to discover that acute T-cell functional exhaustion could be caused by acute SARS-CoV-2 infection, giving rise to the fact that elderly or immunosuppressed patients are more likely to develop severe disease or even death (50). T-cell exhaustion, assumed only in chronic infection or tumor in the past, could also occur in acute infection among those with poor immune homeostasis. No sooner after that had researchers from America and Germany investigated the peripheral blood of patients with COVID-19 with scRNA-seq technology and further verified our discovery (51, 52). Professor Chang Moon wrote the article titled “Fighting COVID-19 exhausts T cells” in *Nature Reviews Immunology* Research Highlight section, thoroughly analyzed our research results, and pointed out that acute T-cell exhaustion is needed to be reversed in COVID-19 treatment; Miriam Merad, a member of American Academy of Arts and Science, wrote reviews in journals such as *Immunity* to affirm our work, considered that our results might be the most important immunological discovery in COVID-19 research field up to now, and pointed out that reversing acute T-cell depletion might be applied in severe COVID-19 treatment. Our discovery was also written into the pathogenesis part in “Guidelines for diagnosis, treatment, and prevention of coronavirus disease 2019 in Chinese adults”, which provides important foundations for diagnosing, preventing, and treating COVID-19 (5). Up to December 2022, this article was directly cited 2012 times (Google Scholar) and was selected for the Science Citation Index high-cited paper. Recently, a retrospective analysis by a German group also discovered that 13 patients with melanoma who received PD-1 monoclonal antibody therapy were mild or asymptomatic after SARS-CoV-2 infection (18), which shows that reversing acute T-cell exhaustion is effective and safe and could be applied in treating critical COVID-19.

### 4.2 The first in the world to put forward that increasing thymic output could rescue “acute T-cell exhaustion”

Since the human thymus begins to shrink at 10~12 years, for elderly patients over 80 years old, the thymic output function is lost. Their capability of antiviral infection mainly depends on the immune homeostasis of peripheral T cells, which is challenging to maintain a 6- to ~8-week antiviral response. How to reverse acute T-cell function exhaustion? On the basis of previous studies, our team proposed a scheme that uses thymosin α1 (Tα1) to promote thymic output and rescue acute T-cell exhaustion. In a cohort of 77 patients with severe COVID-19, after 7 days of consecutive injection of Tα1, thymic output and T-cell counts were significantly increased, and T-cell exhaustion was reversed in elderly patients with CD8^+^ T cells less than 400/μl and CD4^+^ T cells less than 650/μl; correspondingly, the usage of invasive and noninvasive oxygen therapy was lowered, and the mortality rate of critical patients was significantly decreased (53). This strategy that enhances the temporary output of immune cells from central immune organs to increase the homeostasis of peripheral immune systems could rescue acute T-cell exhaustion, is effective in severe COVID-19 treatment, and could also be applied in other critical illnesses. After publication, this article was recommended as a reading article for COVID-19 treatment in a global community.

### 4.3 We discovered that SARS-CoV-2 could directly invade secondary lymphoid organs and kidney

It is already known that SARS-CoV-2 infects cell types expressing ACE2 and TMPRSS2, such as airway goblet cells, ciliated columnar cells, type II alveolar cells, and intestinal epithelial cells. Could SARS-CoV-2 also attack the secondary lymphoid organ that is the base camp of human immunity? SARS-CoV-2 RNA was observed in the bronchoalveolar lavage fluid and the peripheral blood T cells and B cells through scRNA-seq technology by some authors. However, there is no direct evidence that SARS-CoV-2 could infect T cells, B cells, or other immune cells (54). We examined the autopsy tissue and discovered that SARS-CoV-2 could directly infect secondary lymphoid organs in severe or dead patients, such as the spleen and lymphoid nodes. It could be seen in the spleen and lymphoid nodes that ACE2 was mainly expressed in macrophages and dendritic cells. This infection pathway leads to the secretion of inflammatory factors such as IL-6 and TNF-α in infected cells, which causes necrosis and apoptosis of immune cells and, further, the quantity reduction of lymphocytes (55).

**Figure 2:**
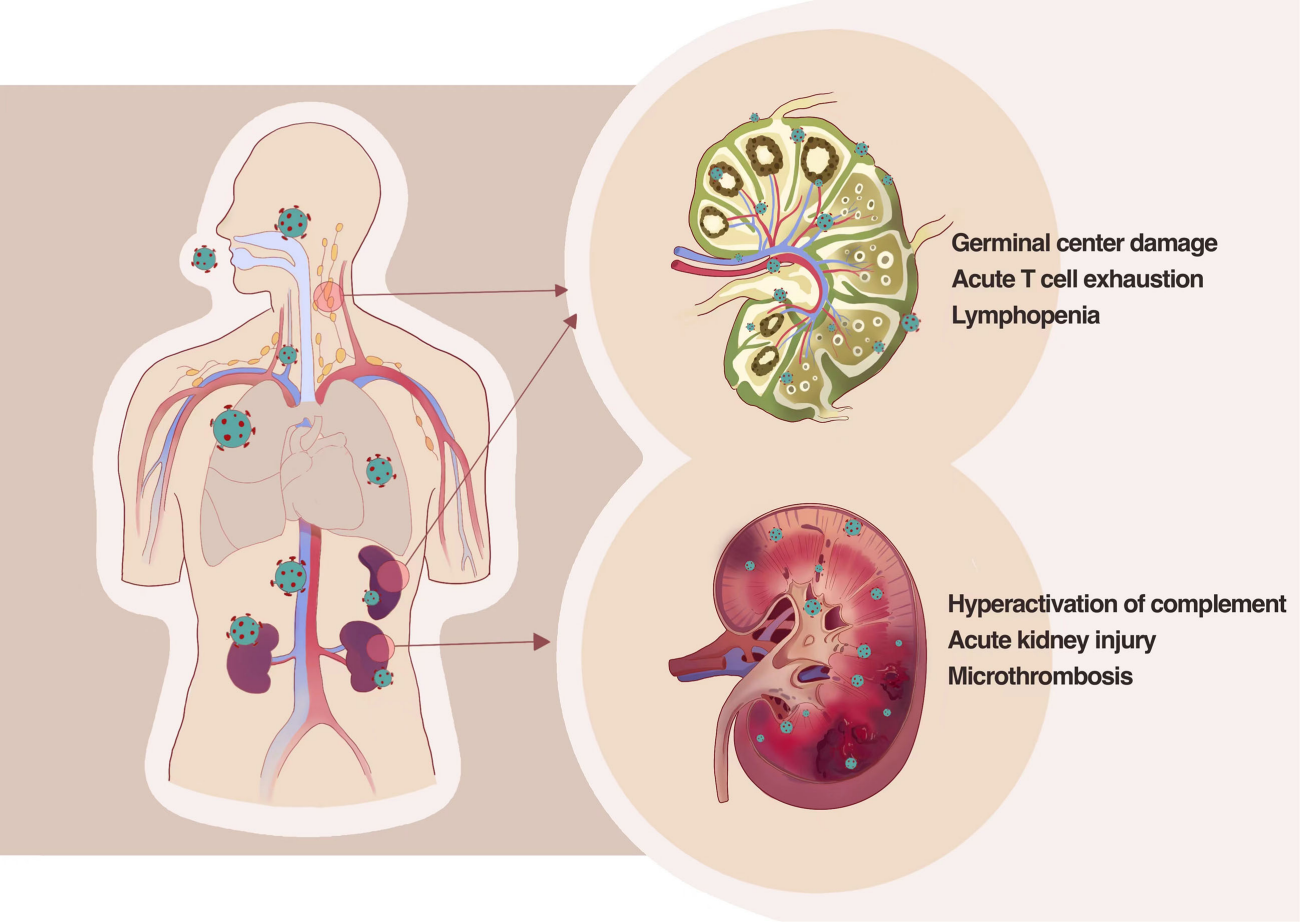
SARS-CoV-2 directly infects the human kidney and the second lymphoid tissues, including lymph nodes and spleens, by thus deteriorating tissue damage. SARS-CoV-2 could directly infect secondary lymphoid organs in severe or dead patients leading to the secretion of inflammatory factors such as IL-6 and TNF-α in infected cells, which causes necrosis and apoptosis of immune cells and further the quantity reduction of lymphocytes. Pathological examination of kidney tissue from patients with COVID-19 and autopsy revealed that SARS-CoV-2 directly invade human kidney through ACE2 receptor and mediate acute kidney injury and severe tubular injury.

Kidney is an organ that highly expresses ACE2 and TMPRSS2. Our group has been focused on whether the kidney gets involved in SARS-CoV-2 infection since the early stages of the pandemic. Clinical observation discovered that proteinuria and hematuria are common manifestations among hospitalized patients with COVID-19; further pathological examination of kidney tissue from patients with COVID-19 and autopsy has been made, which revealed that SARS-CoV-2 directly invade human kidney through ACE2 receptor and mediate acute kidney injury; severe tubular injury could be seen in pathological sections of kidney from autopsy tissue in patients with COVID-19; there are many viral inclusion bodies, i.e., virus N antigen particles in tubular epithelial cells, along with infiltration of a large number of lymphocytes and deposition of complement complexes, which lead to the immune damage of kidney (56), as shown in **Figure 2**.

### 4.4 Invention of COVID-19 antigen diagnostic test kit

Early discovery, diagnosis, and treatment are crucial in combating the COVID-19 pandemic. Antigen-antibody–specific reactions could be applied in the diagnosis of SARS-CoV-2 infection. Because the median seroconversion of antibodies would not be accomplished until 18~20 days after exposure to the SARS-CoV-2 virus on the body, early diagnosis could not be achieved utilizing antigen-antibody reaction. In cooperation with Zhu Jiang Hospital and Southern Medical University clinical laboratory and firms, we developed a rapid and convenient method based on fluorescence immunochromatographic assay to detect the SARS-CoV-2 NP antigen in March 2020. By studying the polymerase chain reaction (PCR) test of nasopharyngeal swab specimens from 253 patients in Wuhan, it was proved that the accuracy of this method was 100%; the specificity was 99%, with simple operations and short turnaround time of 10 min; and the diagnosis could be made 1 day before the onset of the symptom. This method also acquired the technology criteria of diagnosis of WHO (57), accompanied by the certificates of many countries such as China and the European Union.

## 5 Prospects

SARS-CoV-2 has caused over 600 million documented cases around the globe, and more than 6 million deaths, which brings paramount losses to human health, society, and economic development. Most governments worldwide tightened containment measures at the initial stage of the COVID-19 epidemic, including introducing screening at ports of entry, quarantine for infected people, closure of public places for gathering, mass SARS-CoV-2 molecular and serological screening, and lockdown at the state or even national level, which have played an essential role in combatting COVID-19. However, with the gradual liberalization of the pandemic worldwide, mobility and activity restrictions have been gradually removed. Personal protection, such as vaccination, mask-wearing, hand hygiene, and physical distancing, is still strongly advocated to limit the spread of COVID-19.

Coronavirus infection could be a disease as crucial as influenza. Fifteen new untreatable virus diseases have emerged in recent 20 years; in the future, new and emerging infectious diseases remain the challenges to be dealt with in economic and social security. Immuno-diagnosis, immuno-prophylaxis, and immune therapy are fundamental strategies for preventing and controlling diseases of this kind, and clarifying the mechanism of immune damage and protection is the basis for developing these high-quality methods. Meanwhile, back feeding the vaccinology based on frontiers in modern immunology becomes the theme of the ongoing third vaccine revolution. On the one hand, immunology is a cutting-edge discipline in anti-epidemic. It should enter the forefront of anti-epidemic the first time the epidemic occurs to clarify the mechanism of immune damage and protection as soon as possible to provide foundations in immune diagnosis, immune-prophylaxis, and immune treatment. On the other hand, technologies and methods in the immunological study should be closer to actual practice. It is imperative to innovate such technologies and methods in scientific study, product development, and evaluation, which is fast, efficient, practical, and more suitable for the population. Since the outbreak of the epidemic, our team was the first to initiate the research on the coronavirus universal vaccine, which covers the SARS-CoV-2 and its main variants, SARS-COV and other coronaviruses already discovered in bats. On the basis of the latest scientific knowledge in the mechanisms of immune protection and immune damage, we adopted the theory and technology system on protein antigen engineering, which was originally developed by our team, as well as technologies in big data, immune informatics, and molecular design that applied to *de novo* design the immunogen of a universal vaccine, aimed at critical nodes such as a combination of virus and its receptor, virus and cell membrane, protective humoral immunity, protective cellular immunity, mucosal immunity, and mutation of virus, which could escape the immunological surveillance; we designed and screened the immunogen of a universal vaccine, which received the excellent outcome in animal experiments using new adjuvant and nasal drug delivery system. Furthermore, targeting the hyper-inflammatory response in patients with severe COVID-19, we prepared a complete set of cytokine antibodies, such as anti–IL-6, anti–IL-8, anti–IL-10, anti–TNF-α, and anti-MCAF, which could stop inflammatory damage, and anti–TGF-β which prevent pulmonary fibrosis. These works above form the technology reserves in preventing and controlling the pandemic.

## Author contributions

YS and BL write this manuscript. YC and YW revised it. All authors contributed to the article and approved the submitted version.
